# Clinical Neuropathology image 1-2017: incidental schwannoma of the posterior root 

**DOI:** 10.5414/NP301010

**Published:** 2016-12-14

**Authors:** Ellen Gelpi, Mikel Vicente-Pascual

**Affiliations:** 1 Neurological Tissue Bank of the Biobanc-Hospital Clinic-IDIBAPS, Barcelona, and; 2 Neurology Department, Hospital Clinic de Barcelona, Spain

**Keywords:** schwannoma, incidental, posterior roots

## Abstract

No Abstract available.

We describe the incidental postmortem findings in an 83-year-old female brain donor. She had a history of stroke, from which she recovered ad integrum. The brain showed mild Alzheimer’s disease changes with a mild to moderate density of neuritic plaques, capillary amyloid angiopathy, mild τ-positive neurofibrillary pathology (A2, B1, C2), and mild age-related temporo-medial astrogliopathy (ARTAG) with subventricular thorn-shaped astrocytes. 

At the level of the thoracic spinal cord there was a small, well delineated nodular lesion within the posterior root (Figure 1A) in an excentric position. The lesion was composed of elongated tumor cells with fusiform and tapering nuclei and elongated cytoplasmic processes, growing in a herringbone-like pattern, consistent with a small schwannoma. Tumor cells were strongly immunoreactive for S100. 

Schwannomas, also known as neurinomas or neurilemmoma, are homogeneous and slowly growing benign tumors of the nerve sheath, originating from Schwann cells [1]. They occur outside (e.g., skin and subcutaneous tissue) and inside the central and peripheral nervous system. Symptoms depend on their location, radiculopathy being the most frequent initial symptom followed by paresthesia and limb weakness [2]. Peripheral schwannomas may present as asymptomatic tumors, may be incidentally found on imaging studies, and few cases may be discovered at postmortem. They represent 8% of intracranial tumors, 85% of cerebellar angle tumors, and 29% of spinal nerve root tumors [3]. Spinal schwannomas have an incidence of 0.3 – 0.4 cases per 100,000 persons per year. The majority shows predilection for sensory nerves and 75% appear in sensory dorsal roots. Approximately 90% are solitary and sporadic lesions, 4% develop in the setting of neurofibromatosis type 2 (NF2), and 5% are multiple but not associated with NF2 [2]. 

## Conflict of interest 

The authors report no conflict of interest. 

**Figure 1.**
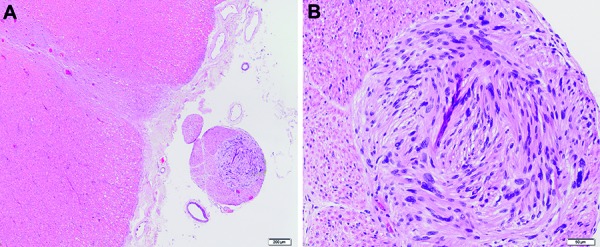
Section at the level of the thoracic spinal cord showing a small, excentric nodular lesion within the posterior root (A). B: Higher magnification.
